# P-225. Polypharmacy, Drug-Drug Interactions, and Risk of Hospitalization among Veterans with HIV Taking 2-, 3-, and 4-Drug Antiretroviral Therapy Regimens

**DOI:** 10.1093/ofid/ofaf695.447

**Published:** 2026-01-11

**Authors:** Lei Yan, Cassidy Henegar, Kei-Hoi Cheung, Vincent Marconi, Maria C Rodriguez-Barradas, Leigh Ragone, Bryn Jones, Vani Vannappagari, Amy Justice, Mihaela Aslan

**Affiliations:** Veterans Affairs VA Connecticut Healthcare System, Cooperative Studies Program Clinical Epidemiology Research Center CSP-CERC, New Haven, Connecticut; ViiV Healthcare, Chapel Hill, North Carolina; Veterans Affairs VA Connecticut Healthcare System, Cooperative Studies Program Clinical Epidemiology Research Center CSP-CERC, New Haven, Connecticut; Emory University, Atlanta, Georgia; Michael E. DeBakey VAMC and Baylor College of Medicine, Houston, Texas; ViiV Healthcare, Chapel Hill, North Carolina; ViiV Healthcare, Chapel Hill, North Carolina; ViiV Healthcare, Chapel Hill, North Carolina; Yale School of Medicine, West Haven, CT; VA/Yale, West Haven, Connecticut

## Abstract

**Background:**

As people with HIV (PWH) age while on antiretrovirals (ARVs), they experience greater non-ARV polypharmacy, increasing their risk of drug-drug interactions. Using data on PWH in the Veterans Aging Cohort Study (VACS) in the US, we assessed whether non-ARV medication count and overlapping prescriptions with known pairwise drug interactions (KPDIs) were associated with risk of hospitalization.

**Figure:**
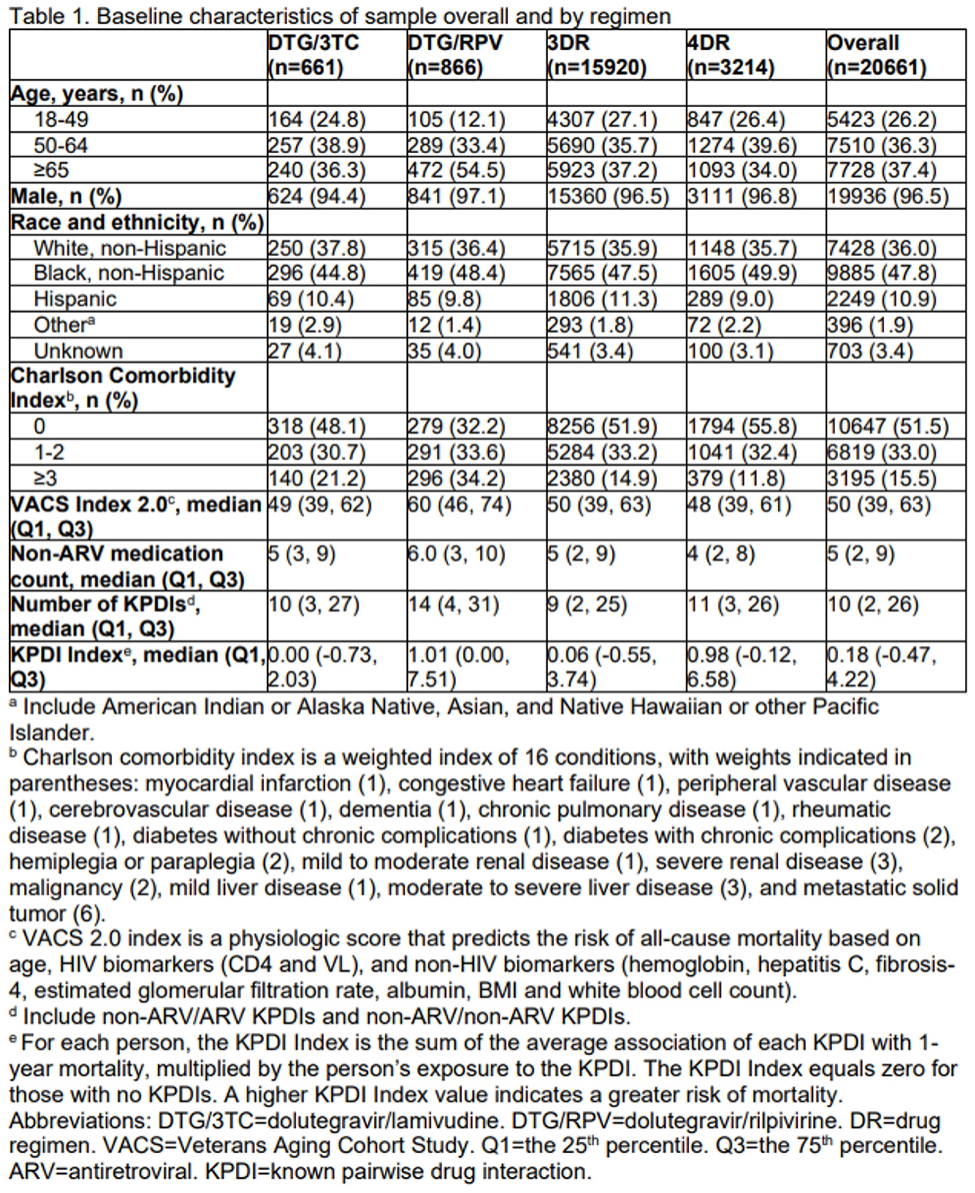

**Figure:**
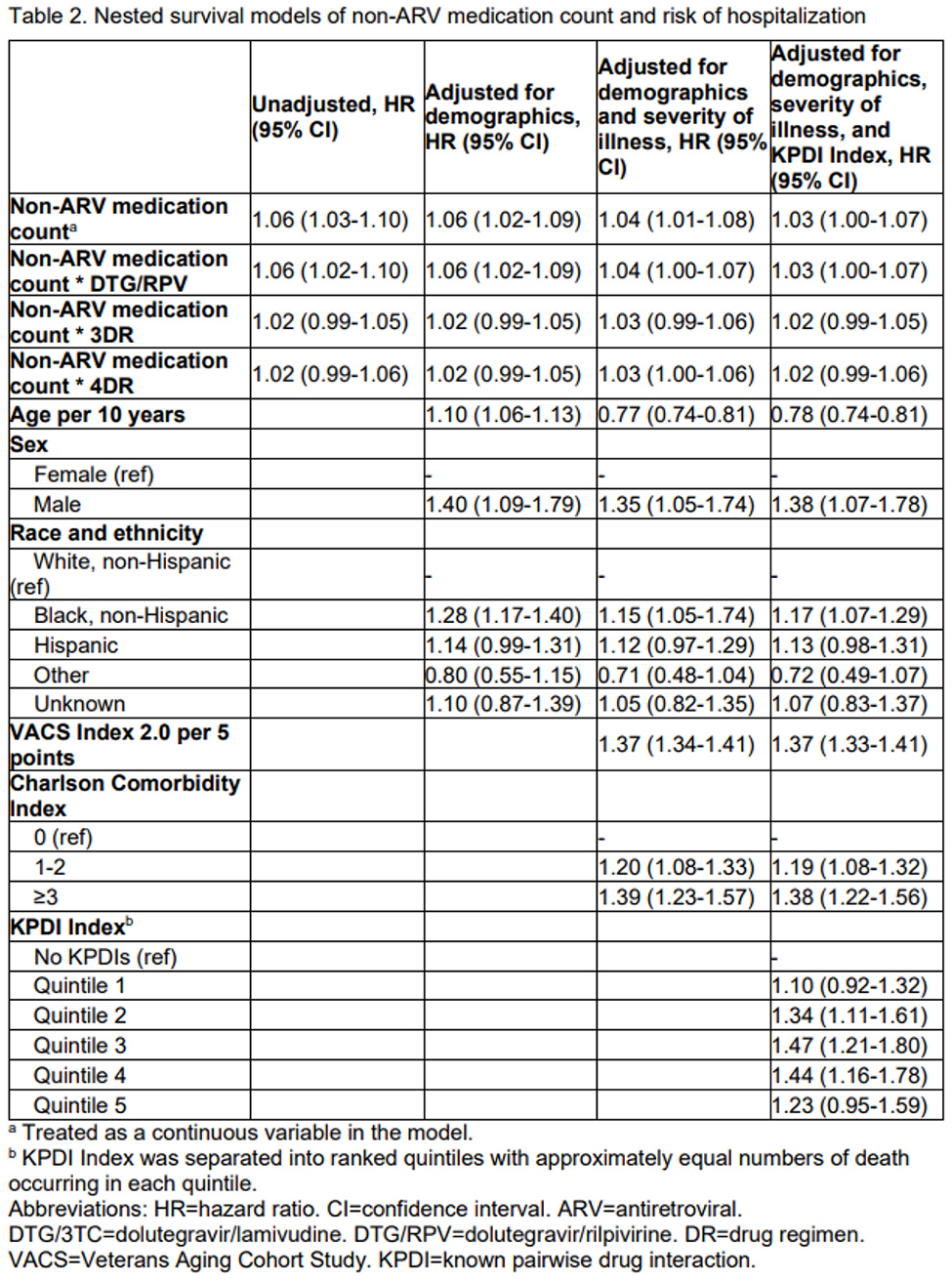

**Methods:**

We included PWH enrolled in the VACS taking 2-drug ARV regimens [2DR; dolutegravir/lamivudine (DTG/3TC), dolutegravir/rilpivirine (DTG/RPV)], 3DR (integrase inhibitor, protease inhibitor, or non-nucleoside reverse transcriptase inhibitor with 2 nucleoside reverse transcriptase inhibitors), or 4DR (3DR plus cobicistat or ritonavir) between 01OCT2020 and 30SEP2023. Pharmacy fill records were used to identify all non-ARV prescriptions overlapping during the course of the baseline ARV regimen. Pairs of medications with KPDI were identified from the DrugBank database. We constructed an exposure-weighted KPDI Index using the average association of each identified KPDI with 1-year mortality. We used nested Cox models to estimate the association between non-ARV medication count and 1-year risk of hospitalization, adjusting for KPDI Index.

**Results:**

The analysis included 20,661 PWH (DTG/3TC: 661, DTG/RPV: 866, 3DR: 15,920, 4DR: 3,214), with 97% male, 36% White, 48% Black, and 11% Hispanic [Table 1]. Those on DTG/RPV were older, had more comorbidities, and higher VACS Index 2.0 scores. Median [IQR] non-ARV medication count ranged from 4 [2, 9] for 4DR to 6 [3, 10] for DTG/RPV. Median KPDI Index ranged from 0.26 [-1.07 to 2.69] for DTG/3TC to 1.81 [-0.54 to 8.11] for 4DR. Higher non-ARV medication count was associated with increased risk of hospitalization regardless of ARV regimen, and adjusting for demographics, frailty, and KPDI Index reduced these associations [Table 2]. Generally, those with higher KPDI Index had greater risk of hospitalization.

**Conclusion:**

The association between increasing non-ARV medication count and risk of hospitalization may be partially explained by drug-drug interactions.

**Disclosures:**

Cassidy Henegar, PhD, MSPH, ViiV Healthcare: Employee|ViiV Healthcare: Stocks/Bonds (Public Company) Vincent Marconi, MD, Lilly: Grant/Research Support|Merck: Grant/Research Support Leigh Ragone, MS, GSK: Stocks/Bonds (Private Company)|ViiV Healthcare: Employee Bryn Jones, MBChB, GSK: Stocks/Bonds (Public Company)|ViiV Healthcare: Employee Vani Vannappagari, MBBS, MPH, PhD, ViiV Healthcare: Full time Employee of ViiV Healthcare and owns GSK stock|ViiV Healthcare: Stocks/Bonds (Public Company)

